# Improved Learning Gain in Medical Students by Using Animated Whiteboard-Videos in Comparison to Textbooks in Surgery

**DOI:** 10.1177/23821205241262684

**Published:** 2024-06-14

**Authors:** Markus Koch, Simone A. Günster, Anna Widder, Florian Seyfried, Christoph-Thomas Germer, Joy Backhaus, Sarah König, Johan F. Lock

**Affiliations:** 1 Department of Trauma, Hand, Plastic and Reconstructive Surgery, 27207 University of Würzburg, Würzburg, Germany; 2 Department of Pediatric Surgery, University Medical Center Erlangen, Friedrich-Alexander-University Erlangen-Nürnberg, Erlangen, Germany; 3 Department of General, Visceral, Transplant, Vascular and Pediatric Surgery, University Hospital Würzburg, Würzburg, Germany; 4 Institute for Medical Teaching and Medical Educational Research, 27207 University Würzburg, Wurzburg, Germany

**Keywords:** animated video, learning gain, learning behavior, surgical education

## Abstract

**BACKGROUND:**

Animated videos have become popular in teaching medical students, although there is a certain lack of evidence concerning its efficacy. Surgery seems to be an ideal field for its application, since animations are very helpful to understand anatomic structures and complex procedures. The aim of this study was to investigate the effects of animated videos compared to textbooks on learning gain.

**METHODS:**

A prospective 2-arm cohort study with 5th-year medical students was conducted during their 2-week surgical training module. The initial cohort of students received textbook sections on 3 major topics in visceral surgery as learning medium (text cohort). During the following semester, the second cohort of students received 3 animated whiteboard videos (animated videos) containing equivalent content (video cohort). All participants completed a multiple-choice test consisting of 15 questions on the learning content at baseline (pre-test) and after the learning period (post-test) and answered an additional evaluation questionnaire.

**RESULTS:**

Both cohorts were similar in their descriptive data and demonstrated significant learning gain during the 2-week learning period. The video cohort achieved better results (80% vs 73% correct answers; *P* = .028) and a higher learning gain (17% vs 11%; *P* = .034) in the post-test compared to the text cohort. The estimated learning time was longer in the video cohort (62 min vs 37 min; *P* < .001) and watching the videos resulted in higher learning gain (21% vs 6%; *P* < .001). Subgroups with higher learning gain by video learning were female gender (20% vs 11%; *P* = .040), native German speakers (18% vs 11%; *P* = .009), students without prior surgical experience (19% vs 12%; *P* = .033) and those undecided concerning a surgical career (22% vs 9%; *P* = .020). Interestingly, “low digital orientation” students benefited from videos (22% vs 13%; *P* = .021), whereas “high digital orientation” students did not.

**CONCLUSIONS:**

Animated videos increase medical students’ learning gain and interest in surgery.

## Introduction

Digital media—with a particular focus on learning videos—have emerged in medical education. Learning videos change the teaching and learning processes and have become widely accepted and utilized media.^1–3^ Recent studies have suggested that videos could be successfully integrated into individual and multimodal learning activities of medical students, enabling flexible access to the content concerning both time and location.^1,4–7^ Learning videos have not been intended to replace face-to-face lectures or seminars,^1,8^ but rather to provide additional factual or procedural knowledge at the learner's individual pace.^1,7,9^ Medical students have expressed their preference for audio-visual learning media.^1,10^

However, there has been a lack of evidence whether videos are actually superior in comparison to traditional learning formats like textbooks, and which covariates might moderate their learning outcomes.^4,11–19^ Animated videos enable greater imaging creativity and convey facts in a simplified manner, thus enabling easy understanding of even complex issues, such as surgical procedures. In addition, the didactic style of animated videos can transport knowledge on a more personal and emotional level,^20^ which might be beneficial for the academic and estimated learning gains.^2,21^

The aim of this study was to investigate the effects of using animated whiteboard videos in comparison to textbooks in surgery on the learning gain of medical students.

## Materials and methods

### Study design and participants

This prospective cohort study was conducted at the Medical Faculty of the Julius-Maximilians-Universität (JMU) Würzburg, Germany. All students who participated in the 2-week surgical training module during the 5th-year of the human medicine degree program were included. The human medicine degree program at the JMU is a 6-year curriculum comprising 2 preclinical years, 3 clinical years and a final practical year. In the 5th-year, students complete various clinical trainings. Here, the semester is divided into 6 groups, each consisting of 10 students, who are consecutively assigned to 7 different clinical departments following a rotation plan. At this point in time, the students had already completed the lecture series and bedside teaching in the specialty of surgery.

**Figure 1.**
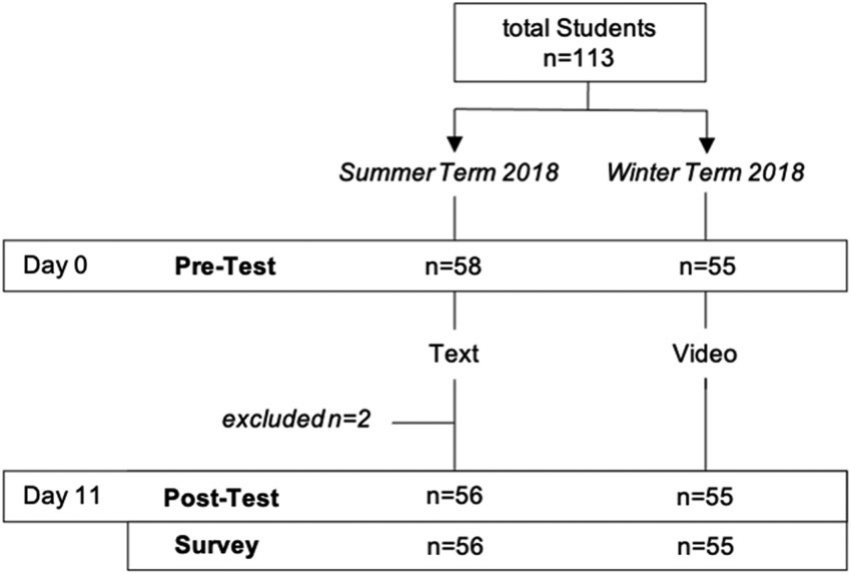
Study design—the first semester received textbook chapters, the second semester had online access to 3 animated whiteboard videos on the same topic.

Students received instructions detailing the study at the beginning of their training module. Study participation was voluntary and non-participation did not affect their course result. Data collection was conducted during the summer term of 2018 and the winter term of 2018. Three major topics from the field of visceral surgery were selected (appendicitis, cholecystitis, and diverticulitis). The study design is presented in Figure 1.

To evaluate and compare the 2 learning formats, the first cohort was provided with 3 textbook chapters covering the selected topics during summer term 2018. The chapters were taken from the 4th edition of the textbook “Duale Reihe Chirurgie” published by Thieme Verlag 2012.^22^ The chapter on appendicitis comprised a total of 8 pages (4.617 words), the chapter on cholecystitis comprised 14 pages (8.293 words), and the chapter on diverticulitis comprised 4 pages (2.624 words). The second cohort was granted online access to 3 animated whiteboard videos during their 2-week surgical training module in winter term 2018.

**Figure 2.**
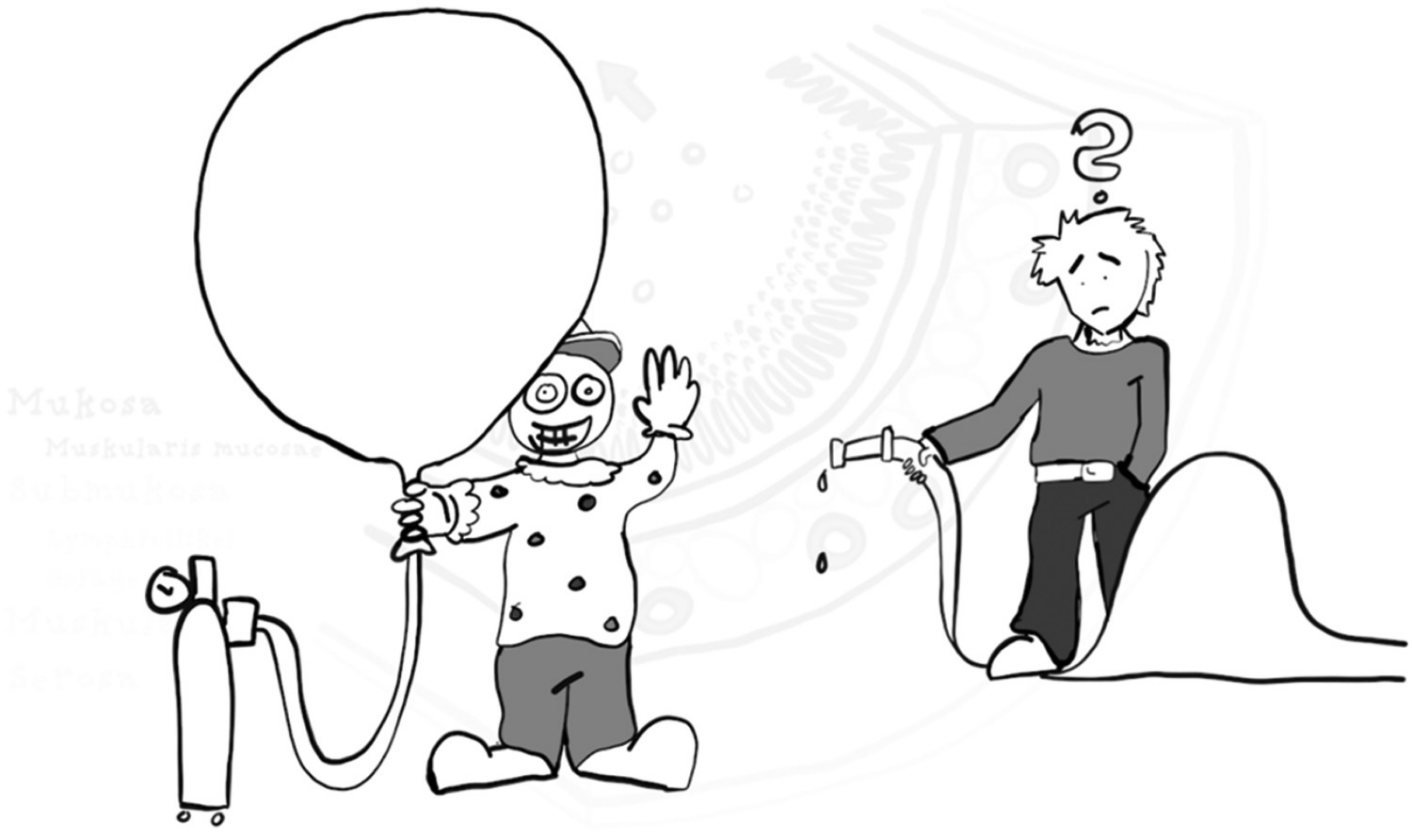
Screenshot of the animated whiteboard video “appendicitis.”

The learning videos were based on whiteboard animations (animated videos).^23^ However, unlike traditional whiteboard animations, the process of drawing was not shown (an example is shown in Figure 2). The production of the videos as well as the scriptwriting were carried out by 2 surgeons with teaching experience and a medical student with technical and design knowledge. Drawings for the videos were made on a Tablet using Adobe Illustrator Draw® (Adobe Inc., San José, USA). The video editing was performed using Adobe Premiere CC® (Adobe Inc., San José, USA). The scripts were recorded using a Sennheiser® microphone (Sennheiser GmbH & Co. KG, Wedemark-Wennebostel, Germany) and MetaRecord® (Apogee Inc., California, USA). The videos were exported in Quick-Time format using H.264 codec at a resolution of 1920 × 1080 pixels. The audio track was recorded at 44.1 kHz in AAC format. The length of each video was about 20 min. For easier access to the specific topics within the videos, each topic was exported separately as a separate sequence and indexed accordingly. After the pre-test on the first day of the surgical training module, either the texts or the videos were made available to the participating students through the university's learning platform “WueCampus.” A download function of the videos was not available.

At the end of the data collection, the videos were published on the e-learning website “mysurgery” (https://www.mysurgery.de/erklaervideos/).

### Assessment of learning gain

The learning gain was analyzed comparing the pre-test level (day 0) with the post-test at the end of the 2-week training (day 11). Both tests consisted of the same questions presented in a single best answer format. The constructive alignment of learning objectives and assessment was ensured for both, the videos and the textbook chapters. The order of the 15 questions was randomized individually in both tests. Participants were given 15 min to complete the test. The tests were administered and evaluated using the web-based program CaseTrain (JMU Würzburg, Germany). Learning gain was calculated in the percentage of correct answers between the post-test and the pre-test.

### Evaluation of participants

In addition, a 51-item evaluation survey was developed for this study and provided to participants through EvaSys® (Version 7.1, 2018, Electric Paper Evaluationssysteme GmbH, Lüneburg, Germany). The survey included items related to demographic characteristics, learning behavior, individual learning preferences, digital affinity and evaluation of the learning media text and video. Mostly the items using a 5-point Likert scale ranging from (1) Strongly disagree, (2) Disagree, (3) Neither agree nor disagree, (4) Agree to (5) Strongly agree. The mean results of Likert scale (M) were reported. Some items were binary, such as the question about gender or native language German. Not indicating a preference was always a possible response.

In the video cohort, students additionally rated 11 items related to technical aspects and the individual acceptance of the videos. Some of the items were adopted from a previously published study by Backhaus et al which compared vodcasts with traditional lecture.^13^

### Statistical analysis

The results are presented as mean values, unless stated otherwise. Learning gain was individually calculated as the difference of the test results (post-test–pre-test divided by pre-test [%]). The learning outcomes were compared using either paired or independent t-test, as appropriate. The Likert scale questions were analyzed using Mann–Whitney U-test. Statistical significance was assumed at a *P*-value of <.05 and marked with *, while high significance at *P* < .01 was marked width ** and very high significance at *P* < .001 was marked with ***. Statistical analyses were performed using SPSS® (Version 25, 2017, IBM Corporation).

The subgroups “Digital orientation” and “Video affinity” were calculated from the evaluation questionnaires using Spearman's coefficients. Average high or low levels of agreement of respective items were used to assign students within subgroups, see Table 1. The subgroup “Digital orientation” was determined from the evaluation question asking whether medical students were familiar with internet-enabled devices and used them in many parts of their daily lives. “Video affinity” was determined based on the items asking whether students used videos as a serious source of information in private or for study. Allocation to the subgroup “full use of media” was based on the affirmative response to whether the medium in question was fully read or watched, respectively.

**Table 1.** Assignment to the subgroups.

SUBGROUP	MEASUREMENT	MEAN^a^	ASSIGNMENT
Digital orientation	5 Items (A1-A5)^b^	< 3	Low digital orientated
		≥ 3	High digital orientated
Video affinity	5 Items (B1-B5)^b^	< 3	Low video affinity
		≥ 3	High video affinity
Use of media	3 Items (C1-C3)^b^	< 3	Less use of media
		≥ 3	Full use of media

^a^Average value on a 5-point Likert scale for the respective items.

b See Appendix.

## Results

A total of 111 out of 113 medical students in the surgical training participated in this study and provided pre-test, post-test, and evaluation forms. The descriptive statistics of the students are provided in Table 2.

**Table 2.** Characteristics of students and learning activity.

	**TEXT**	**VIDEO**
*n* =	56	55
Age [mean (range)]	25 (22-36)	25 (23-36)
Female gender [% (*n*)]	63 (35)	55 (30)
Native language German [% (*n*)]	84 (47)	95 (52)
Previous surgical experience [% (*n*)]	21 (12)	22 (12)
Interest in surgical career [% (*n*)]	23 (13)	24 (13)
High digital orientated [% (*n*)]	47 (27)	53 (31)
High video affinity [% (*n*)]	43 (33)	57 (44)
Learning activity		
Read/watched the full media^a^ [M]	3.0	4.2 **
Estimated learning time [min]	37	62 **

M, means of 5-point Likert scale; n, sample size; min, minutes.

^a^Reading the text versus watching the videos.

** *P* < .01.

The students answered several questions in the survey regarding their digital affinity and learning preferences: Medical students reported using computers, tablets, and smartphones in many aspects of everyday life (M [Mean Likert scale] 4.3) and felt confident in their ability to operate them well (M 4.2). The majority of students described themselves as fairly technology affine (M 3.4) and enjoyed watching movies (M 3.9). Concerning their learning preferences, students preferred self-determining their learning pace (M 4.4), visual illustrations (M 4.2), and moving pictures (M 3.6). Students rated learning videos as an efficient way to obtain information and to learn (M 3.7).

### Learning gain

**Figure 3.**
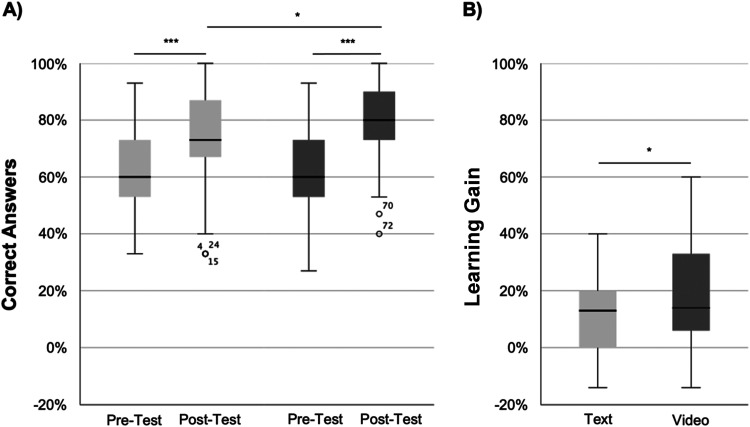
Comparison of learning gain between text and video cohort. (A) Pre- and post-test results of student performance; (B) Overall learning gain.

No difference in baseline knowledge (pre-test) was detected between the text- and video cohort ([correct answers] 62% vs 63%; *P* = .813). Both cohorts demonstrated significant learning gain in the post-test. The video cohort achieved better results (80% vs 73%; *P* = .028), as well as learning gain in the post-test (17% vs 11%; *P* = .034; see Figure 3).

* *P* < .05; *** *P* < .001.

The overall learning gain was not influenced by age, gender (15% vs 13%; *P* = .444), native German speakers (15% vs 10%; *P* = .323), previous surgical experience (15% vs 9%; *P* = .086), or interest in a surgical career (14% vs 13%; *P* = .661). However, learning with videos significantly influenced superior learning gain in the subgroups displayed in Table 3. Specifically, female students, students without previous surgical experience and students undecided about a surgical career had a higher learning gain. Interestingly, high video affinity supported learning gain through videos, whereas high digital orientation did not. In addition, students who watched the entire videos performed better.

**Table 3.** Learning gain (in %) within subgroups.

	TEXT	VIDEO	*P-*VALUE
Female	11%	20%	.040*
Native-German	11%	18%	.009*
No previous surgical experience	12%	19%	.033*
Undecided for surgical career	9%	22%	.020*
Low digital orientation	13%	22%	.021*
High video affinity	8%	18%	.002*
*Watched videos completely*		21%	
*Watched videos not completely*		6%	.008*

*P < .05.

### Evaluation of learning media

Textbooks were considered as useful additions to lectures (M 4.2), as were animated videos (M 4.4). However, neither textbooks (M 2.5) nor videos (M 2.4) could fully replace them. Both media, textbooks (M 4.3), and videos (M 4.5) allow flexible knowledge transfer in terms of time and place. Students generally prefer having more videos in their studies (M 4.1).

Participants in the video cohort spent significantly longer time using their learning media than participants in the text cohort (62 min vs 37 min; *P* = .001). Only 36% of students actually read the provided textbook chapters completely, while 64% watched all provided videos.

The video cohort also rated their learning medium more favorably than the text cohort. For example, the videos focused more on specific content, the presentation was more structured and made relationships clearer. Furthermore, interest in visceral surgery was more enhanced by videos than by textbooks.

**Figure 4.**
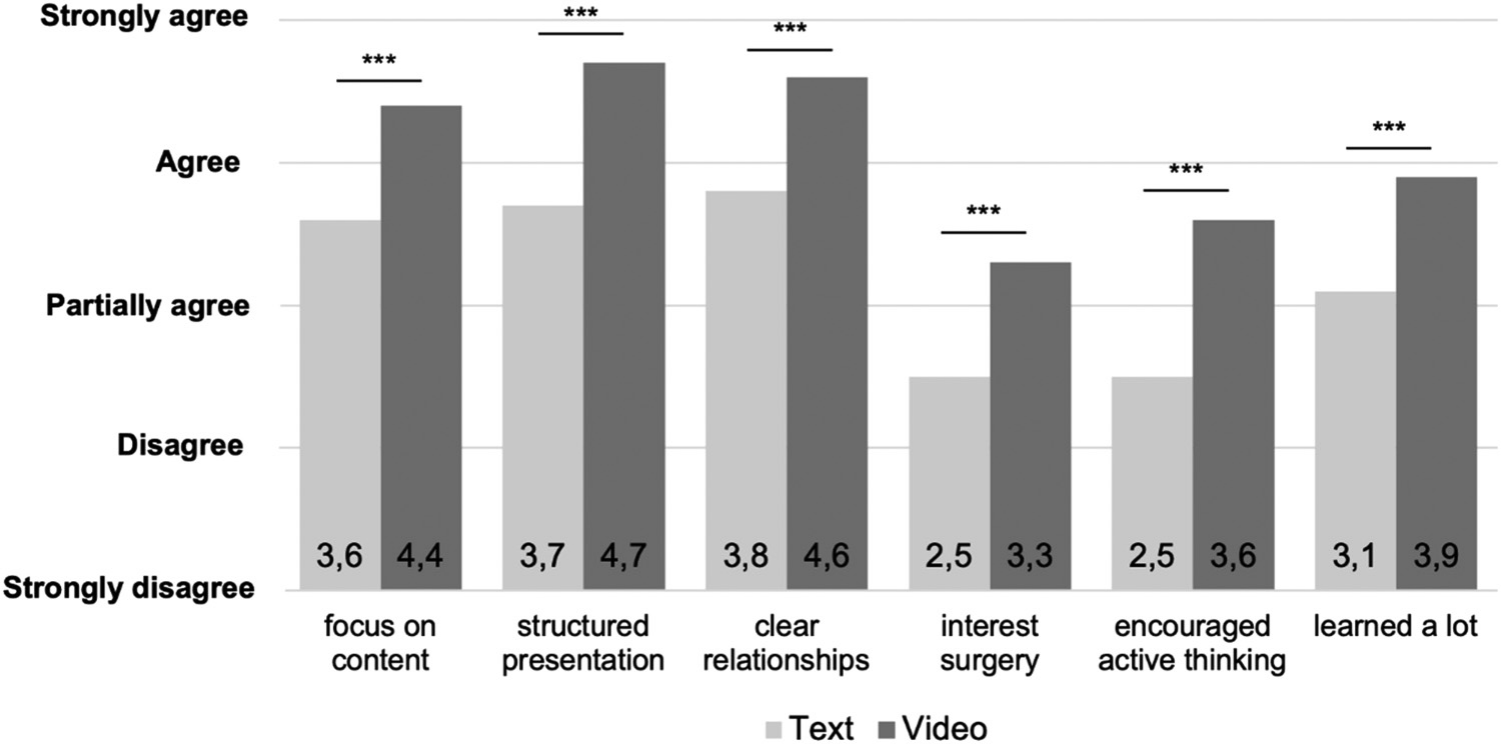
Students’ evaluation of the media. The data are expressed as means on a 5-point Likert scale.

Students who participated in the video cohort felt more encouraged to think actively than those in the text cohort. Finally, the feeling of having learned a lot was significantly greater in the video cohort, see Figure 4.

*** *P* < .001.

### Evaluation of the animated videos

The structural design was rated most positively (M 4.7), followed by the technical implementation (M 4.5) and the didactic quality (M 4.5). Furthermore, the layout of the videos was rated positively (M 4.4). The students found the videos to be entertaining (M 3.9). The length of the videos, which were approximately 20 min long, was considered appropriate by most participants. Only a few students experienced technical difficulties, mostly related to problems starting the videos or short interruptions during the playback.

## Discussion

Growing digitalization and the Covid-19 pandemic, as a kind of booster, have rapidly shifted learning formats from classic face-to-face to digital alternatives. Digital teaching methods, such as e-learning websites, apps, and virtual reality, have garnered increasing interest from both students and teachers. Videos, in particular, have become a focus, with several subcategories, each having unique characteristics. Within in this highly relevant context, the present study proposes the adoption of animated whiteboard videos as an additional digital teaching tool in visceral surgery, as well as for the entire medical degree.

The majority of the published literature focused on video formats such as recorded lectures, tutorial videos, or screencasts. However, no superiority over conventional learning media such as lectures or textbooks has been demonstrated in most studies.^4,11,13,15,18,19^ There is one randomized trial available that showed superior learning gain by podcasts versus textbooks in orthopaedics.^24^

In contrast to previous studies, this study specifically focuses on the use of animated videos compared to textbooks in surgical training. Interestingly, there has been little examination of animated videos as a teaching tool. The study was conducted over 2 consecutive semesters to increase the study population and avoid potential confounding factors from the use of other learning media. The corresponding learning material, local environment (eg, rooms used) and time frame were controlled and kept the same in both groups to minimize further potential confounding factors. Both groups were homogeneous in terms of socio-demographic characteristics and represented an entire semester.

Our findings showed a significant learning gain in both groups. However, students in the video cohort had significantly better results than those in the text cohort. Furthermore, the subjective learning gain was also reported in favor of the video learning. It should be emphasized that animated videos encouraged more active thinking and conveyed a sense that participants had learned a lot.

The students of both groups were digitally and technically affine. The results of our study indicate that the use of laptops, tablets, and smartphones has already become prevalent and consistent in their daily lives. Thus, the prerequisite for utilizing digital educational offerings is not a lack of knowledge in handling these devices, but rather, the availability of easy technical access and adequate information about the available resources. Students particularly value the ability to independently access knowledge anytime and anywhere. These positive effects of utilizing videos have already been published in the literature.^4–6^ Both texts and videos were consumed in a quiet and familiar environment. Similar to previously published studies, participants rated videos as being equally useful as textbooks, although neither format was seen as a substitute for conventional lectures.^1,8^ Accordingly, animated videos represent a valuable supplement to the curriculum in human medicine.

To distinguish our results further, we categorized the participants into the following dichotomous groups: those with “high digital orientation” versus “low digital orientation,” and “high video affinity” versus “low video affinity.” Among the “low digital orientation” students, the group that watched animated videos showed the greatest objective and subjective learning gain. Therefore, the increased learning gain from watching animated videos is not related to excessive digital affinity. On the other hand, “high video affinity” students achieved better results when watching videos, while “low video affinity” students performed better when reading textbooks. Students are capable of effectively evaluating teaching materials that suit them best. Thus, different teaching media affect students differently and student-centered teaching should focus on providing various teaching formats that fit to individual students’ needs.

Notably, participants in the video cohort dedicated more time to their medium compared to the textbook cohort. This is indicative that videos serve as an effective means to sustain prolonged motivation for engaging with a topic. Through this heightened engagement, further positive influences can be inferred, like the following. Prolonged use of the medium enhances knowledge gain, a finding corroborated by the present data.

We also demonstrated how the videos can increase students’ interest in visceral surgery. This is particularly noteworthy among female students who represent the majority of students and the future surgeons. Our study is of exceptional relevance, especially considering that only 23% of the participants were interested in a surgical career, and there is a shortage of young surgeons in German-speaking countries.

The production of animated whiteboard videos can be time-consuming and requires certain technical prerequisites. However, these videos can have long-term benefits and promote individual learning gain among students. At present, animated videos are not a common teaching strategy due to their complex technical implementation, unlike videos with PowerPoint-slides and voice-overs. Nonetheless, they can be a useful addition to the curriculum, especially for specific topics. We recommend discussing the video content using the flipped classroom concept and addressing any open questions. Alternatively, a digital platform can be established for students to ask questions. When creating animated videos, it is important to consider the didactic implementations, even in online teaching, since the attention span of students is typically no more than 15 to 20 min.

## Limitations

Due to the lack of data we were unable to perform a prior sample size calculation. In addition, the evaluation questionnaires of learning media were not previously validated.

Only 2 semesters containing a relatively small number of participants were analyzed. Although no differences in socio-demographic parameters were found, confounders such as differences in socioeconomic and educational background cannot be fully excluded. Likewise, a detection bias may have had an influence on the results, especially on the media evaluation. Participants in the video cohort, who were aware of being allowed to try out a novel learning method, may have evaluated it more positively. The increased time spent on learning in the video cohort resulted also from the fact that the 60 min were required to fully watch the videos (3 pieces of 20 min each).

No additional follow-up to evaluate a potential long-term difference in learning outcomes was performed.

## Conclusions

Animated whiteboard videos could have a positive impact on learning in surgical education of medical students. Watching animated videos led to a significantly greater objective and subjective learning gain than reading textbooks in this study. In particular female students and students without prior surgical experience benefit most from video-based learning in surgery.

## Appendix

### Items

A1—“I am an individual who is enthusiastic about technology.”

A2—“I am proficient in operating tablets and smartphones.”

A3—“I enjoy reading digitally.”

A4—“I use computers, tablets, and smartphones in many aspects of my daily life.”

A5—“I tend to use images more frequently than words to express myself.”

B1—“I prefer watching videos over reading texts.”

B2—“Videos are an efficient means for me to acquire information.”

B3—“I can better retain content through videos than through written texts.”

B4—“I regularly watch videos on medical topics.”

B5—“The instructional videos were very helpful for me in preparing for the OSCE.”

C1—“I have fully read the text/watched the video on the topic of ‘cholecystitis’.”

C2—“I have fully read the text/watched the video on the topic of ‘appendicitis’.”

C3—“I have fully read the text/watched the video on the topic of ‘diverticulitis’.”
